# Impact of Laparoscopic Nissen Fundoplication on Non-complicated Barrett’s Esophagus

**DOI:** 10.4103/1319-3767.80381

**Published:** 2011

**Authors:** Ashraf A. Mohamed, Khaled M. Mahran, Mohamed M. Zaazou

**Affiliations:** Minia University Hospital, Minia, Egypt; 1 Misr University for Science and Technology (MUST) Hospital, 6^th^ of October City, Egypt

**Keywords:** Barrett’s esophagus, esophageal dysplasia, fundoplication

## Abstract

**Background/Aim:**

Laparoscopic fundoplication can alter the natural course of Barrett’s esophagus (BE). This study was undertaken to assess this role in patients with non-complicated BE.

**Materials and Methods:**

From October 2004 to October 2009, 43 patients with BE (32 men and 11 women) underwent laparoscopic Nissen fundoplication surgery in the Department of Surgery at Minia University Hospital. The median age of these patients was 46 years (range: 22–68 years). Patients with high-grade dysplasia, invasive cancer, or previous antireflux surgery were excluded. All 43 patients had gastroesophageal reflux symptoms. Heartburn was present in all patients, regurgitation in 41 (95.3%), dysphagia in 8 (18.6%), retrosternal pain in 30 (69.8%), upper gastrointestinal hemorrhage in 6 (13.9%), and respiratory symptoms in 19 (44.2%). Nissen fundoplication was performed in all patients. Thirty-four patients (79.1%) had concomitant hiatal hernia and nine patients (20.9%) had low-grade dysplasia.

**Results:**

The median follow-up period was 25.6 months. There was significant improvement of symptoms after surgery (*P*<0.05). Eight (18.6%) of those with short-segment BE had total regression and four (9.3%) of those with long-segment BE had a decrease in total length. Among the nine patients with preoperative low-grade dysplasia, dysplasia disappeared in seven, remained unchanged in one, and progressed to in situ adenocarcinoma in one patient.

**Conclusions:**

laparoscopic fundoplication succeeded in controlling symptoms but had unpredictable effect on dysplasia and regression of BE. Laparoscopic fundoplication does not eliminate the risk of developing esophageal adenocarcinoma and therefore, endoscopic follow-up should be continued in these patients.

Barrett’s esophagus (BE) is a condition defined by columnar appearing mucosa of any length in the distal esophagus on endoscopic examination that shows specialized intestinal metaplasia on histology.[1] The definition of BE varies between countries, with the presence of goblet cells required for a diagnosis of BE in all countries except in the UK and Japan.[2] BE is considered a complication of excessive reflux, which may cause esophagitis and impairment of esophageal peristalsis[3] which, in turn, prolongs contact of the refluxate with the esophagus and enhances mucosal damage. Patients with gastroesophageal reflux disease (GERD) show variable endoscopic findings, ranging from a normal esophagus to ulcerative esophagitis to BE.[4] About 5%–15% of patients with chronic GERD have BE.[5,6] Because of its malignant potential, BE is dealt with caution by gastroenterologists and surgeons.[7] It is associated with increased risk of esophageal adenocarcinoma,[8] with one study reporting that the risk increases about 50-fold.[9] Laparoscopic fundoplication has been proven to be an effective operation for dealing with medically refractory GERD.[10–13] As regard its indications and results in patients with BE, controversy still exists.[14] Some studies have reported regression of esophageal intestinal metaplasia with fundoplication.[9,15] However, patients with BE often have more severe reflux symptoms, with potential risk for sequelae such as strictures, presbyesophagus, and esophagitis, so the clinical outcomes in such patients after laparoscopic Nissen fundoplication is not always satisfactory.[16,17] The aim of this study was to evaluate the clinical outcome and the histopathologic regression of BE after antireflux surgery.

## MATERIALS AND METHODS

### Patient population

A total of 43 patients with symptomatic BE were seen in the Department of Surgery at Minia University Hospital between January 2001 and January 2006. BE had been confirmed on at least two upper endoscopies with biopsy before treatment and these patients also had two or more upper endoscopies with biopsy after treatment. There were 32 male and 11 female patients, with a median age of 46 years (range: 22–68 years). Patients with high-grade dysplasia, invasive cancer, or previous antireflux surgery were excluded. All patients underwent a laparoscopic Nissen fundoplication. Median follow-up was 25.6 months. All patients were evaluated by a detailed history, which included information regarding use of antacids or any acid-reducing medication and the presence or absence of typical and atypical GERD symptoms.

### Endoscopic features and histopathology

At each endoscopy, the location of the gastroesophageal junction was defined as the point where the tubular esophagus meets the proximal extent of the gastric rugal folds. The extent of Barrett’s epithelium was measured from this point to the highest point of the squamocolumnar junction. A hiatal hernia was diagnosed when the crural impression was separated from the top of the gastric rugal folds by 2 or more centimeters. Four-quadrant biopsies were taken from the columnar mucosa at 2-cm intervals, with histopathologic examination of specimens after hematoxylin and eosin staining. The diagnosis of BE was confirmed by identification of specialized intestinal metaplasia on at least two pretreatment endoscopies.

### Study definitions

When the length of columnar epithelium containing specialized intestinal epithelium was <3 cm it was classified as short-segment Barrett’s esophagus (SSBE), and when the length was ≥3 cm it was termed long-segment Barrett’s esophagus (LSBE). Histopathologic regression was defined as disappearance of intestinal metaplasia or regression from low-grade dysplasia to specialized intestinal metaplasia.

### Operative technique

Laparoscopic fundoplication was carried out in the standard fashion as described by Soper.[18] Briefly, the fundoplication was constructed around a 15-mm Savary dilator after posterior crural closure with interrupted non-absorbable sutures. The short gastric vessels were divided to allow full fundic mobilization. Then, a 2-cm wrap was created with three interrupted non-absorbable sutures.

### Postoperative care

For the first 24 hours after surgery, patients were administered intravenous metoclopramide and ketorolac to reduce the risk of postoperative emesis and to minimize pain. Nasogastric tubes were removed and clear liquids were allowed the morning after surgery, with advancement to a soft diet later that day, followed by discharge from the hospital. Postoperative clinical assessment, including questioning regarding GERD-related symptoms and medication use, was performed at 1 month, at 6–12 months, and annually thereafter. Surveillance endoscopy was performed for all patients at 1-year follow-up after operation. The chi-square test and Student’s t test were used for statistical analysis; *P*≤0.05 was considered significant. Summary data are presented as mean±SD or as percentages.

## RESULTS

The study subjects included 32 men and 11 women. The median age at the time of surgery was 46 years (range: 22–68 years). All patients underwent preoperative esophagogastroduodenoscopy and all had biopsy-proven BE. The BE was circumferential in 24 patients (55.8%) and patchy in 18 (41.9%). The median length of BE was 3 cm (range: 2–12 cm). Lengths greater than or equal to 3 cm (LSBE) were found in 22 patients (51.2%). Other concomitant findings included sliding hiatal hernia in 34 patients (79.1%) (diagnosed by barium swallow) and low-grade dysplasia in 9 patients (20.9%).

The main clinical features of the 43 patients with BE are shown in Table 1.

**Table 1:** Clinical features of patients

	Patients
Median age (year)	46
Sex (M/F)	32/11
Duration of symptoms in months (mean±SD)	62±21
Heartburn (No., %)	43 (100)
Regurgitation (No., %)	41 (95.3)
Dysphagia (No., %)	8 (18.6)
Upper gastrointestinal hemorrhage (No., %)	6 (13.9)
Retrosternal pain (No., %)	30 (69.8)
Respiratory symptoms (No., %)	19 (44.2)

All patients were managed initially with a medical regimen that consisted of lifestyle and dietary modifications, proton pump inhibitors, H _2_ -blockers, and antacids. The median duration of medical management before surgery was 5 years (range: 2–14 years).

The most common indication for surgery [Table 2] was the presence of symptoms refractory to medical therapy (42 patients, 97.7%).

**Table 2:** Indications for surgery in patients with Barrett’s esophagus

Indications	Number (%)
Refractory symptoms	42 (97.7)
Low-grade dysplasia	9 (20.9)
Sliding hiatal hernia	34 (79.1)

Postoperative esophagogastroduodenoscopy and biopsy were performed for all patients at 1-year follow-up after surgery. BE was absent in eight patients (18.6%) of those with SSBE and had decreased in length by greater than 2 cm in four patients (9.3%) of those with LSBE. Additional endoscopic findings included esophageal narrowing that required dilatation in one patient (2.3%). Of the nine patients with low-grade dysplasia, complete regression of the dysplasia to nondysplastic Barrett’s occurred in seven cases (77.8%); this was significantly more common in SSBE than in LSBE, occurring in 5 of 21 (23.8%) and 2 of 22 (9.1%) patients, respectively (*P*=0.01). Progression to *in situ* adenocarcinoma occurred in one patient (11.1%) (LSBE; at 23 months), and there was no change in one patient (11.1%) who was treated with photodynamic therapy. The patient with *in situ* adenocarcinoma subsequently underwent esophageal resection. This patient is currently alive and has been free of disease over the period of follow-up after esophagectomy. For the remaining 21 patients, no change has occurred.

With laparoscopic fundoplication, there was improvement in the symptoms of gastroesophageal reflux in patients with BE [Table 3].

**Table 3:** Response of symptoms after surgery

	Preoperative	Postoperative	*P* value
Heartburn (No., %)	43 (100)	4 (9.3)	<0.001
Regurgitation (No., %)	41 (95.3)	3 (6.9)	<0.001
Dysphagia (No., %)	8 (18.6)	1 (2.3)	0.01
Upper gastrointestinal hemorrhage (No., %)	6 (13.9)	0	<0.001
Retrosternal pain (No., %)	30 (69.8)	2 (4.6)	
Respiratory symptoms (No., %)	19 (44.2)	0	

## DISCUSSION

The incidence of esophageal adenocarcinoma is increasing in the United States, thus highlighting the significance of BE, a premalignant lesion. Longer segments of Barrett’s indicate longer duration of gastroesophageal reflux. So, patients with LSBE have higher risk for developing malignancy.[19] In the current study, progression to *in situ* adenocarcinoma occurred in one patient (11.1%) and this was one of those with LSBE. Currently, most clinicians initially treat BE and its associated symptoms with proton pump inhibitors which may need to be continued for prolonged periods.[20] Trastek[20] considered refractory symptoms an indication for surgical intervention, and this was the indication in all but one of our patients. Regression of BE did occur in our study. Eight patients (18.6%) had total regression and four patients (9.3%) had partial regression. In addition, regression of low-grade dysplasia to no dysplasia occurred in seven of nine patients. Although regression of BE following antireflux surgery has occasionally been reported in the past,[21–25] a number of recently published studies have also demonstrated complete regression.[5,26–28] Regression remains an unpredictable event as the factors responsible for its occurrence have not yet been determined.[28] Laparoscopic fundoplication controlled symptoms in the majority of patients with BE in a study by Abbas *et al*.[14] This was true in the current study also, with the symptoms being significantly controlled after surgery (*P*<0.05).

In conclusion, laparoscopic fundoplication succeeded in controlling symptoms in the majority of patients with BE. However, it is not yet possible to predict in which patient disappearance of BE and reversal of dysplasia may occur. Laparoscopic fundoplication does not eliminate the risk of developing esophageal adenocarcinoma. Therefore, endoscopic follow-up should be continued in these patients.
